# Associations of audiometric hearing and speech-in-noise performance with cognitive decline among older adults: The Baltimore Longitudinal Study of Aging (BLSA)

**DOI:** 10.3389/fneur.2022.1029851

**Published:** 2022-12-09

**Authors:** Kening Jiang, Nicole M. Armstrong, Yuri Agrawal, Alden L. Gross, Jennifer A. Schrack, Frank R. Lin, Luigi Ferrucci, Susan M. Resnick, Jennifer A. Deal, Danielle S. Powell

**Affiliations:** ^1^Cochlear Center for Hearing and Public Health, Johns Hopkins Bloomberg School of Public Health, Baltimore, MD, United States; ^2^Department of Epidemiology, Johns Hopkins Bloomberg School of Public Health, Baltimore, MD, United States; ^3^Department of Psychiatry and Human Behavior, Warren Alpert Medical School, Brown University, Providence, RI, United States; ^4^Intramural Research Program, National Institute on Aging, Baltimore, MD, United States; ^5^Department of Otolaryngology-Head and Neck Surgery, Johns Hopkins University School of Medicine, Baltimore, MD, United States; ^6^Center on Aging and Health, Johns Hopkins University, Baltimore, MD, United States; ^7^Department of Health Policy and Management, Johns Hopkins Bloomberg School of Public Health, Baltimore, MD, United States

**Keywords:** aging, hearing loss, audiometry, speech perception, cognition

## Abstract

**Background:**

Established associations between hearing loss and cognitive decline were primarily defined by pure-tone audiometry, which reflects peripheral hearing ability. Speech-in-noise performance, which reflects central hearing ability, is more limited in prior literature. We examined the longitudinal associations of audiometric hearing and speech-in-noise performance with cognitive decline.

**Methods:**

We studied 702 participants aged ≥60 years in the Baltimore Longitudinal Study of Aging 2012–2019. Global and domain-specific (language, memory, attention, executive function, visuospatial ability) cognitive performance were assessed by the cognitive assessment battery. Hearing thresholds at 0.5, 1, 2, and 4 kilohertz obtained from pure-tone audiometry were averaged to calculate better-ear pure-tone average (PTA) and participants were categorized as having hearing loss (>25 decibels hearing level [dB HL]) or normal hearing (≤25 dB HL). Speech-in-noise performance was assessed by the Quick Speech-in-Noise (QuickSIN) test, and participants were categorized as having below-median (worse) or above-median performance. Linear mixed effects models with random intercepts and slopes were used to assess baseline cognitive performance and cognitive decline by hearing status. Models adjusted for demographic, lifestyle and disease factors.

**Results:**

Participants with audiometric hearing loss showed similar baseline cognitive performance but faster decline in global cognitive function, language, executive function, and attention. Participants with below-median QuickSIN score showed worse baseline cognitive performance in all domains and faster decline in global cognitive function, language, memory, executive function and attention.

**Conclusions:**

Audiometric hearing might be targeted to delay cognitive decline. Speech-in-noise performance might be a novel marker and might be more sensitive to memory decline.

## Introduction

Approximately 6.2 million U.S. older adults currently live with Alzheimer's disease and related dementias and this number is expected to rise with population aging (1). Living with cognitive impairment poses challenges for older adults, their families, and societies. Despite the complexity of underlying pathologies and the unmodifiable nature of certain pivotal dementia risk factors like age, emerging evidence indicates that many modifiable factors, including hearing loss, are important in the prevention of dementia (2).

Hearing loss, as defined by pure-tone audiometry, is common among older adults: its prevalence increases from 45% among adults in their 60s to nearly 90% among adults 80 years and older (3). Audiometric hearing loss has been identified as the largest potentially modifiable risk factor for dementia, accounting for up to 8% of dementia cases (2). Previous studies have reported both cross-sectional associations between hearing loss and poorer cognitive performance (4, 5) and longitudinal associations between hearing loss and accelerated cognitive decline among U.S. older adults (6–9). Plausible mechanisms have been proposed linking hearing loss and cognitive decline, including increased cognitive processing effort, structural and functional changes in the brain, and social isolation (10). However, studies in population-based cohorts with pure-tone audiometry and longer follow-up periods are needed to further clarify the associations and understand how cognitive domains might be differentially affected.

Although pure-tone audiometry is the gold standard for hearing evaluation that has long been used for defining hearing loss, pure-tone audiometry is primarily a measure of peripheral auditory function, reflective of the initial encoding of auditory signals. Other hearing evaluations like speech-in-noise tests, which characterize central auditory function (the decoding of auditory signals), instead rely on higher-level cognitive processing. Difficulties understanding speech in the presence of noise are common among older adults and significantly impact daily living. Cognitive functions like working memory and attention have been related to speech-in-noise performance (11), suggesting that speech-in-noise performance might be a marker for cognitive deficits. However, prior evidence investigating speech-in-noise performance and cognitive decline in large population-based cohorts is limited (12, 13).

To bridge the gaps in longitudinal evidence that examines and compares the impacts of peripheral and central aspects of hearing on cognition in well-established aging cohorts, this study aims to investigate associations of both audiometric hearing and speech-in-noise performance with global and domain-specific (language, memory, attention, executive function, visuospatial ability) cognitive decline over follow-up among adults aged 60 years and older using data from the Baltimore Longitudinal Study of Aging (BLSA). We hypothesize that worse audiometric hearing and speech-in-noise performance are both associated with accelerated rates of cognitive decline.

## Materials and methods

### Study population

The Baltimore Longitudinal Study of Aging (BLSA) is an ongoing cohort study of aging conducted by the National Institute on Aging since 1958. BLSA enrolls community-dwelling healthy U.S. adults aged 20 years and older continuously. Enrolled participants are followed for life. Participants undergo comprehensive health assessments during study visits every 1–4 years depending on age (20–59 years: every 4 years; 60–79 years: every 2 years; ≥80 years: every year). Details of the BLSA design have been published previously (14, 15). Written informed consent was obtained from all participants and the Institutional Review Board of the Intramural Research Program approved the study protocol.

Hearing evaluations were performed in the BLSA from 2012 onwards. This study included BLSA participants aged 60 years and older with hearing and cognitive measures between 2012 and 2019. We identified 738 participants aged 60 years or older with complete data on pure-tone audiometry and speech-in-noise performance at ≥1 study visit and defined their first visit as the baseline in this analysis. We further excluded 3 participants missing all cognitive assessments and 33 participants with missing data on covariates, leaving a final analytical sample of 702 participants.

### Cognitive performance

Global and domain-specific cognitive performance, as our outcomes of interest, were assessed by a battery of neurocognitive tests at each study visit between 2012 and 2019. Descriptions of the cognitive tests are presented in Table 1. A total of five cognitive domains were constructed using factor analysis methods:

(1) Language was represented by Letter Fluency Test (16), Category Fluency Test (16), and Boston Naming Test (17);
(2) Memory was represented by immediate and long-delay free recall from the California Verbal Learning Test (18);
(3) Attention was represented by Trail Making Test Part A (19) and Digit Span Forward Test (20);
(4) Executive function was represented by Trail Making Test Part B (19), Digit Span Backward Test (20), and Digit Symbol Substitution Test (21);
(5) Visuospatial ability was represented by Card Rotations Test (22) and Benton Visual Retention Test (23).

**Table 1:** Description of the cognitive tests in the Baltimore Longitudinal Study of Aging 2012–2019.

**Domain**	**Cognitive test**	**Interpretation**
Language	Letter fluency test	Number of words generated in 60 s
	Category fluency test	Number of words generated in 60 s
	Boston naming test	Number of pictures identified
Memory	California verbal learning test immediate recall	Number of words recalled
	California verbal learning test long-delay recall	Number of words recalled
Attention	Trail making test part A	Time to completion (seconds)
	Digit span forward test	Maximum length of digits recalled
Executive function	Trail making test part B	Time to completion (seconds)
	Digit span backward test	Maximum length of digits recalled
	Digit symbol substitution test	Number of symbol/digit pairs completed in 90 s
Visuospatial ability	Card rotations test	Number classified correctly—number classified incorrectly
	Benton visual retention test	Number of errors

Global cognitive performance was represented by all the tests mentioned above. Scores from Benton Visual Retention Test and Trail Making Test Parts A and B were reversed (multiply by −1) so that higher scores for all the cognitive tests reflect better performance. Individual cognitive tests were standardized by converting to *Z* scores using the baseline mean and standard deviation (SD) for comparison across tests. Corresponding standardized test scores in each cognitive domain were then used to derive global and domain-specific cognitive factor scores using structural equation modeling for confirmatory factor analysis, where observed covariation in the manifest variables (cognitive test scores) was explained by the latent variables (cognitive factor scores) (24, 25).

Global cognitive factor score served as our primary outcome of interest and five domain-specific cognitive factor scores (language, memory, attention, executive function, visuospatial ability) served as our secondary outcomes of interest.

### Hearing measures

Pure-tone audiometry, as our measure of peripheral auditory function, was conducted using Interacoustics AD629 audiometer with ER3A insert earphones in a sound-attenuating booth according to best-practice procedures (26). Participants were presented with pure-tone signals at frequencies between 0.5 and 8 kilohertz (kHz) and were instructed to raise hands when they heard the signal. The intensities of the signals were decreased until the participants no longer responded to determine the quietest sound participants indicated they heard the signal (hearing threshold) at each frequency. Air-conduction hearing thresholds in each ear were obtained and expressed in decibels hearing level (dB HL). Pure-tone average (PTA) was calculated by averaging hearing thresholds at 0.5, 1, 2, and 4 kHz for each ear, higher PTA indicates worse audiometric hearing. PTA in the better-hearing ear was analyzed continuously (per 10 dB HL worse) and categorically comparing participants having hearing loss (PTA >25 dB HL) to those with normal hearing (≤25 dB HL) according to common clinical cut-points (27).

The Quick Speech-in-Noise (QuickSIN) test assesses participants' ability to understand speech in the presence of background noise (28). Participants were presented with two lists of six sentences at a fixed presentation level (70 dB HL) first in quiet and then under successively higher levels of background noise. Participants were instructed to repeat as much of the sentences as they could. Each sentence has five target words and the scoring of the test is based on the correct identification of target words. Scores of two lists were averaged to represent mean number of target words correctly identified, ranging from 0 to 30. These raw QuickSIN scores (higher = better performance) were used directly for analysis instead of converting to signal-to-noise ratio (SNR) loss as in clinical settings, which lacks age-specific norms for performance and interpretability in statistical analysis. QuickSIN score was analyzed continuously as per 5-point worse and categorically (below vs. above median) based on statistical distribution in our study population.

### Other covariates

Demographic information was collected *via* self-report, including age (continuous in years), sex (Male; Female), race (White; Black; Other [Combining American Indian or Alaska Native, Chinese, Filipino, Japanese, Other Asian or Pacific Islander, Other non-White, and Not classifiable]) and years of education. Education was categorized as high school or less (≤12 years), any college (12–16 years) and beyond college (>16 years). Self-reported smoking status was collected as current, former and never and was combined as ever/never smoker for analysis due to the small number of participants identified as current smokers. Body mass index (BMI) was calculated from measured height and weight and was analyzed continuously in kg/m^2^. Hypertensive status was defined based on measured systolic blood pressure (SBP) and diastolic blood pressure (DBP): Hypertension was considered present if SBP was ≥140 mmHg or DBP ≥90 mmHg; Prehypertension was considered present if SBP was between 120 and 139 mmHg or DBP was between 80 and 89 mmHg (29). Diabetes was defined as glycated hemoglobin (HbA1c) ≥6.5%. Elevated cholesterol was defined as having total cholesterol ≥200 mg/dL (30). The 20-item Center for Epidemiological Studies-Depression Scale (CES-D) was used to assess depressive symptoms. Participants were asked to report the frequency of each symptom (0: Rarely or none of the time; 1: Some or little of the time; 2: Moderately or much of the time; and 3: Most or almost all the time) and responses were summed to yield total CES-D score (31). Total scores range from 0 to 60 and higher scores indicate greater level of depressive symptoms. All the covariates were defined at the baseline visit.

### Statistical analysis

Baseline characteristics of the participants were summarized and compared by hearing status (Better-ear PTA >25 vs. ≤25 dB HL and QuickSIN score below vs. above median) using ANOVA for continuous variables and Pearson's chi-squared test for categorical variables.

Linear mixed effects models with random intercepts, random slopes, unstructured covariance matrix and robust standard errors were fitted to estimate the longitudinal associations between hearing status at baseline and longitudinal trajectories of cognitive performance. To test whether rates of change in cognitive performance vary by baseline hearing status, models included an interaction term between time since baseline and hearing status. Model assumptions were checked using residual diagnostic plots. For each cognitive factor score (global, language, memory, attention, executive function, visuospatial ability), which is the outcome of interest, we fit separate models for each hearing measure (Better-ear PTA; QuickSIN score) as the main exposure of interest. Non-linear trajectories of cognitive performance were explored by graphical representation and were found for QuickSIN score. We therefore included a liner spline term with a knot at 20 points, which assumes different linear relationships between QuickSIN score and cognitive performance among those with QuickSIN score <20 vs. ≥20 points.

To explore interactions of hearing status with demographic characteristics predicting cognitive decline, 3-way interaction terms (time since baseline × hearing status × age/sex/race) were included in the models to test whether the longitudinal associations between hearing and cognitive performance vary by demographic characteristics. For exploration of interactions, age was categorized as >75 vs. ≤75 years, race was categorized as White vs. Black (excludes 46 participants in other categories), and sex was still analyzed as males vs. females.

Models with better-ear PTA as the main exposure were adjusted for age, sex, race, education, smoking, body mass index, hypertension, diabetes, elevated cholesterol, and depressive symptoms. Models with QuickSIN score as the main exposure were additionally adjusted for better-ear PTA since speech perception in noise depends on the integrity of the auditory signals from the peripheral auditory system.

Analyses were conducted using Stata, version 15.1 (StataCorp LLC, College Station, Texas). A two-sided *P*-value <0.05 was considered statistically significant.

## Results

### Characteristics of the participants

A total of 702 participants (Baseline mean age: 75 years; 45% male; 68% White) were included in our study. Participants were followed for a mean of 3.5 years (Range: 0–7 years) over a mean number of 3 visits (Range: 1–7). Among 702 participants, 258 (37%) had normal audiometric hearing (Better-ear PTA ≤25 dB HL) and above-median (>23) QuickSIN score, 292 (42%) had both audiometric hearing loss (Better-ear PTA >25 dB HL) and below-median (≤23) QuickSIN score, 65 (9%) had normal audiometric hearing but below-median QuickSIN score, and 87 (12%) had audiometric hearing loss but above-median QuickSIN score. 697 of 702 participants had data on hearing aid use and 117 of them had any hearing aid use (6 with normal audiometric hearing and 111 with audiometric hearing loss).

Baseline characteristics of participants by audiometric hearing are presented in Table 2. 379 (54%) participants had audiometric hearing loss and 323 (46%) had normal hearing. When compared to participants with normal hearing, participants with audiometric hearing loss were older (Mean age: 79 vs. 71 years), more likely to be male (54 vs. 35%) and White (78 vs. 55%) and had lower BMI (Mean: 27 vs. 28 kg/m^2^). We also compared baseline characteristics of participants with QuickSIN score below median (worse performance) to those with QuickSIN score above median (Table 3). Participants with QuickSIN score below median were older (Mean age: 79 vs. 71 years), more likely to be male (54 vs. 37%) and White (69 vs. 66%), were less educated (≤High School: 10 vs. 5%), had lower BMI (Mean: 27 vs. 28 kg/m^2^) and greater level of depressive symptoms (Mean CES-D: 5 vs. 4).

**Table 2:** Baseline characteristics of participants by audiometric hearing in the Baltimore Longitudinal Study of Aging 2012–2019.

	**Total *N* = 702**	**Better-ear pure-tone average^a^**		***P*-value^b^**
		**Normal hearing**	**Hearing loss**	
		***N* = 323**	***N* = 379**	
	***N* (%)**	***N* (%)**	***N* (%)**	
Male	317 (45.2)	113 (35.0)	204 (53.8)	<0.001
Race				<0.001
White	474 (67.5)	179 (55.4)	295 (77.8)	
Black	182 (25.9)	120 (37.2)	62 (16.4)	
Other	46 (6.6)	24 (7.4)	22 (5.8)	
Education				0.47
High school or less	50 (7.1)	19 (5.9)	31 (8.2)	
Any college	259 (36.9)	123 (38.1)	136 (35.9)	
Beyond college	393 (56.0)	181 (56.0)	212 (55.9)	
Ever smoker	285 (40.6)	120 (37.2)	165 (43.5)	0.09
Hypertension				0.30
Normal	415 (59.1)	201 (62.2)	214 (56.5)	
Prehypertension	230 (32.8)	98 (30.3)	132 (34.8)	
Hypertension	57 (8.1)	24 (7.4)	33 (8.7)	
Diabetes	98 (14.0)	51 (15.8)	47 (12.4)	0.20
Elevated cholesterol	202 (28.8)	103 (31.9)	99 (26.1)	0.09
	**Mean (SD)**	**Mean (SD)**	**Mean (SD)**	
Age (years)	75.1 (8.3)	70.7 (6.7)	78.7 (7.8)	<0.001
Body mass index (kg/m^2^)	27.3 (4.7)	28.0 (5.0)	26.6 (4.2)	<0.001
CES-D score	4.7 (4.9)	4.6 (4.6)	4.8 (5.1)	0.60
Number of visits	3.0 (1.4)	2.7 (1.1)	3.2 (1.5)	<0.001

SD, standard deviation; CES-D, Center for Epidemiologic Studies Depression Scale.

a Better-ear pure-tone average was calculated using hearing thresholds at 0.5, 1, 2, and 4 kilohertz obtained from pure-tone audiometry. Participants were categorized as having hearing loss (>25 decibels hearing level [dB HL]) or normal hearing (≤25 dB HL).

b P-values were calculated by ANOVA for continuous variables and Pearson chi-squared test for categorical variables.

**Table 3:** Baseline characteristics of participants by speech-in-noise performance in the Baltimore Longitudinal Study of Aging 2012–2019.

	**Total *N* = 702**	**Speech-in-noise performance^a^**		***P*-value^b^**
		**Above median**	**Below median**	
		***N* = 345**	***N* = 357**	
	***N* (%)**	***N* (%)**	***N* (%)**	
Male	317 (45.2)	126 (36.5)	191 (53.5)	<0.001
Race				<0.001
White	474 (67.5)	228 (66.1)	246 (68.9)	
Black	182 (25.9)	105 (30.4)	77 (21.6)	
Other	46 (6.6)	12 (3.5)	34 (9.5)	
Education				0.04
High school or less	50 (7.1)	16 (4.6)	34 (9.5)	
Any college	259 (36.9)	129 (37.4)	130 (36.4)	
Beyond college	393 (56.0)	200 (58.0)	193 (54.1)	
Ever smoker	285 (40.6)	140 (40.6)	145 (40.6)	0.99
Hypertension				0.55
Normal	415 (59.1)	211 (61.2)	204 (57.1)	
Prehypertension	230 (32.8)	108 (31.3)	122 (34.2)	
Hypertension	57 (8.1)	26 (7.5)	31 (8.7)	
Diabetes	98 (14.0)	55 (15.9)	43 (12.0)	0.14
Elevated cholesterol	202 (28.8)	101 (29.3)	101 (28.3)	0.77
	**Mean (SD)**	**Mean (SD)**	**Mean (SD)**	
Age (years)	75.1 (8.3)	70.7 (6.6)	79.2 (7.6)	<0.001
Body mass index (kg/m^2^)	27.3 (4.7)	27.6 (4.8)	26.9 (4.5)	0.04
CES-D score	4.7 (4.9)	4.2 (4.3)	5.2 (5.3)	0.003
Number of visits	3.0 (1.4)	2.7 (1.1)	3.2 (1.5)	<0.001

SD, standard deviation; CES-D, Center for Epidemiologic Studies Depression Scale.

a Total score of the Quick Speech-in-Noise test ranges from 0 to 30. Lower score indicates worse speech-in-noise performance. The continuous score was categorized as below (≤23) vs. above median (>23).

b P-values were calculated by ANOVA for continuous variables and Pearson chi-squared test for categorical variables.

### Associations with audiometric hearing

Baseline cognitive performance comparing participants with audiometric hearing loss to participants with normal audiometric hearing did not differ significantly. However, participants with audiometric hearing loss had faster annual rates of decline in global (Estimate = −0.09 SD, 95% confidence interval [CI]: −0.11, −0.06), language (Estimate = −0.04 SD, 95% CI: −0.06, −0.02), executive function (Estimate = −0.04 SD, 95% CI: −0.07, −0.02) and attention (Estimate = −0.04 SD, 95% CI: −0.07, −0.02) cognitive factor scores when compared to participants with normal hearing (Table 4). Neither group of participants had significant declines in memory domain during follow-up, and no differences in annual rates of decline were observed. Participants in both groups demonstrated significant decline in visuospatial ability, but no differences in rates of decline were found. When PTA was modeled continuously as per 10 dB HL worse, similar results were found.

**Table 4:** Multivariable-adjusted associations^a^ of audiometric hearing with cognitive performance in the Baltimore Longitudinal Study of Aging 2012–2019 (*N* = 702).

**Cognitive factor score^b^**	**Baseline differences: >25 vs. ≤25 dB HL**	**Annual rate of change**			
		**≤25 dB HL (Ref.)**	**>25 dB HL**	**Differences: >25 vs. ≤25 dB HL**	**Differences: Per 10 dB HL Worse**
Global	−0.03 (−0.16, 0.10)	–**0.04** **(**–**0.05**, –**0.03)**	–**0.13** **(**–**0.15**, –**0.10)**	–**0.09** **(**–**0.11**, –**0.06)**	–**0.03** **(**–**0.04**, –**0.02)**
Language	0.02 (−0.09, 0.14)	–**0.02** **(**–**0.03**, –**0.01)**	–**0.06** **(**–**0.07**, –**0.04)**	–**0.04** **(**–**0.06**, –**0.02)**	–**0.01** **(**–**0.02**, –**0.01)**
Memory	−0.04 (−0.18, 0.10)	0.01 (−0.01, 0.03)	−0.02 (−0.04, 0.00)	−0.02 (−0.05, 0.00)	−0.01 (−0.01, 0.00)
Executive function	0.01 (−0.11, 0.12)	–**0.03** **(**–**0.04**, –**0.01)**	–**0.07** **(**–**0.09**, –**0.05)**	–**0.04** **(**–**0.07**, –**0.02)**	–**0.01** **(**–**0.02**, –**0.00)**
Attention	−0.05 (−0.16, 0.06)	−0.01 (−0.02, 0.00)	–**0.05** **(**–**0.07**, –**0.04)**	–**0.04** **(**–**0.07**, –**0.02)**	–**0.02** **(**–**0.03**, –**0.01)**
Visuospatial	0.04 (−0.08, 0.15)	–**0.03** **(**–**0.04**, –**0.02)**	–**0.05** **(**–**0.06**, –**0.03)**	−0.01 (−0.03, 0.00)	−0.00 (−0.01, 0.00)

dB HL, decibels hearing level; CI, confidence interval; Ref, reference.

a Linear mixed effects models with random intercept, random slope, unstructured covariance structure and robust standard errors. Models adjusted for age, sex, race, education, smoking, body mass index, depression, hypertension, diabetes and elevated cholesterol.

b Cognitive test scores were standardized to baseline mean and standard deviation and were used to derive global and domain-specific cognitive factor scores using factor analysis. Lower scores indicate worse performance.

The bold values indicate the value of *p* < 0.05 which are statistically significant.

### Associations with speech-in-noise performance

Participants with QuickSIN scores below the median (worse) showed significantly lower cognitive factor scores at baseline across all domains when compared to participants with QuickSIN scores above the median (Table 5). Participants with QuickSIN scores below the median had faster annual rates of cognitive decline (Global: Estimate = −0.08 SD, 95% CI: −0.11, −0.06; Language: Estimate = −0.03 SD, 95% CI: −0.05, −0.01; Memory: Estimate = −0.03 SD, 95% CI: −0.05, −0.00; Executive function: Estimate = −0.04 SD, 95% CI: −0.06, −0.01; Attention: Estimate = −0.04 SD, 95% CI: −0.06, −0.02). For visuospatial ability, both groups had significant decline during follow-up, but the difference in annual rates of change was not significant. When QuickSIN score was modeled continuously as per 5-point worse, no differences in annual rates of decline were found across all the domains when QuickSIN score is below 20. Significant differences in rates of decline when QuickSIN score is above 20 were similarly observed in global (Estimate = −0.09 SD, 95% CI: −0.13, −0.04), memory (Estimate = −0.04 SD, 95% CI: −0.07, −0.00), executive function (Estimate = −0.04 SD, 95% CI: −0.08, −0.00) and attention (Estimate = −0.04 SD, 95% CI: −0.08, −0.01), while the language domain (Estimate = −0.02 SD, 95% CI: −0.05, 0.00) showed borderline significance (Table 5).

**Table 5:** Multivariable-adjusted associations^a^ of speech-in-noise performance with cognitive performance in the Baltimore Longitudinal Study of Aging 2012–2019 (*N* = 702).

**Cognitive factor score^b^**	**Baseline differences: below vs. above median**	**Annual rate of change**				
		**Above median (Ref.)**	**Below median**	**Differences: below vs. above median**	**Differences: per 5-point worse**	
					**QuickSIN < 20**	**QuickSIN ≥20**
Global	–**0.46** **(**–**0.63**, –**0.29)**	–**0.05** **(**–**0.06**, –**0.03)**	–**0.13** **(**–**0.15**, –**0.10)**	–**0.08** **(**–**0.11**, –**0.06)**	−0.02 (−0.06, 0.02)	–**0.09** **(**–**0.13**, –**0.04)**
Language	–**0.30** **(**–**0.44**, –**0.17)**	–**0.03** **(**–**0.04**, –**0.01)**	–**0.05** **(**–**0.07**, –**0.04)**	–**0.03** **(**–**0.05**, –**0.01)**	−0.01 (−0.03, 0.01)	−0.02 (−0.05, 0.00)
Memory	–**0.23** **(**–**0.40**, –**0.07)**	0.01 (−0.01, 0.03)	−0.02 (−0.04, 0.00)	–**0.03** **(**–**0.05**, –**0.00)**	0.02 (−0.00, 0.04)	–**0.04** **(**–**0.07**, –**0.00)**
Executive function	–**0.36** **(**–**0.51**, –**0.20)**	–**0.04** **(**–**0.05**, –**0.02)**	–**0.07** **(**–**0.09**, –**0.05)**	–**0.04** **(**–**0.06**, –**0.01)**	0.02 (−0.00, 0.04)	–**0.04** **(**–**0.08**, –**0.00)**
Attention	–**0.31** **(**–**0.43**, –**0.19)**	–**0.01** **(**–**0.03**, –**0.00)**	–**0.05** **(**–**0.07**, –**0.03)**	–**0.04** **(**–**0.06**, –**0.02)**	−0.01 (−0.04, 0.01)	–**0.04** **(**–**0.08**, –**0.01)**
Visuospatial	–**0.31** **(**–**0.43**, –**0.18)**	–**0.04** **(**–**0.05**, –**0.03)**	–**0.04** **(**–**0.05**, –**0.02)**	0.00 (−0.01, 0.02)	0.01 (−0.01, 0.03)	−0.00 (−0.03, 0.02)

CI, confidence interval; Ref, reference.

a Linear mixed effects models with random intercept, random slope, unstructured covariance structure and robust standard errors. Models adjusted for age, sex, race, education, smoking, body mass index, depression, hypertension, diabetes, elevated cholesterol and better-ear pure-tone average.

b Cognitive test scores were standardized to baseline mean and standard deviation and were used to derive global and domain-specific cognitive factor scores using factor analysis. Lower scores indicate worse performance.

The bold values indicate the value of *p* < 0.05 which are statistically significant.

### Interaction by demographic characteristics

We did not detect an interaction between hearing status and age (>75 vs. ≤75 years), sex (Males vs. Females) or race (White vs. Black) (Supplementary Figures 1–6). Only the association between QuickSIN score and memory cognitive factor score varied by race, such that differences in rates of change comparing participants with QuickSIN score below the median to those above the median were not significant among White participants (Estimate = −0.01 SD, 95% CI: −0.04, 0.02), but were significant among Black participants (Estimate = −0.08 SD, 95% CI: −0.14, −0.03).

## Discussion

Among 702 BLSA participants aged 60 years or older with up to 7 years of follow-up between 2012 and 2019, after adjusting for demographic, lifestyle and disease factors, we found longitudinal associations of both audiometric hearing and speech-in-noise performance with global and domain-specific cognitive decline. Participants with worse speech-in-noise performance but not audiometric hearing, already showed worse cognitive performance at baseline. Specifically, audiometric hearing loss was associated with faster rates of decline in global cognitive function as well as declines in language, executive function and attention. Participants with speech-in-noise performance below the median (worse) had faster rates of decline in global cognitive function, language, memory, executive function and attention compared to those with scores above the median. In our exploration of interaction, we found an interaction between speech-in-noise performance and race for memory performance, such that the association of speech-in-noise performance with cognitive decline was driven largely by Black participants but not White participants.

Hearing relies on both peripheral and central auditory systems: the peripheral auditory system captures sound signals and converts them into electrical signals in the cochlea; the central auditory system carries these electrical signals to the brainstem and cortex for recognition, integration and understanding of the information. As the gold standard, pure-tone audiometry is widely used in prior literature to define hearing loss and is recognized as a risk factor for cognitive decline and dementia (2). However, pure-tone audiometry does not assess all functions of the hearing system. Using an umbrella term “hearing loss” based solely on the pure-tone audiometry is not sufficient for characterizing the associations. Speech-in-noise performance involves the interplay of peripheral auditory input, central auditory processing, and cognitive functions and is reflective of brain health. Additionally, the speech-in-noise test, as a quick and clinically useful tool, is a vital component of auditory assessment. Therefore, it is important to incorporate multiple aspects of hearing to clarify potential differential associations with cognitive decline and inform hearing health care.

Though inconclusive, prior evidence has documented associations of both worse audiometric hearing and speech-in-noise performance with smaller brain volumes and worse white matter integrity cross-sectionally (32–34) as well as greater brain atrophy and decline in white matter integrity longitudinally (35, 36). The brain structures impacted are directly or indirectly involved in auditory processing, but some also play a role in cognitive processes. The observed associations might thus be explained causally by the reduced neural stimulation of the brain structures for auditory processing caused by degraded auditory signals (10, 35). With the extensive involvement of brain regions and higher-order cognitive processing, speech-in-noise performance is more likely to be a marker instead of a risk factor for cognitive decline.

Our finding that participants with normal hearing and hearing loss, as defined by pure-tone audiometry, had similar cognitive performance at baseline was consistent with the cross-sectional finding of a previous study among 313 participants in BLSA using study years 2012–2015 (9), but was inconsistent with a smaller cross-sectional study among 347 participants in BLSA using years 1990–1994 where a cross-sectional association between worse audiometric hearing and poorer cognitive function was found (5). This could be explained by characteristics of the participants, where the BLSA 1990–1994 cohort was younger (Mean age: 71 years) and had higher proportion of males (65%) and White participants (93%). As with previous longitudinal analyses, our study again demonstrates that audiometric hearing loss is associated with a faster rate of cognitive decline (6–9). Notably, although previous studies showed accelerated decline in the memory domain (6, 9), our study showed borderline differences in rates of decline comparing the hearing loss group to normal hearing group (Estimate = −0.02 SD, 95% CI: −0.05, 0.00, *P* = 0.08). It is possible that we were underpowered to detect a difference in memory decline, given our overall sample is healthy older adults.

Our study found associations between worse speech-in-noise performance and worse cognitive performance at baseline as well as faster decline in cognitive performance over time. These significant associations between speech-in-noise performance and cognition remained robust after adjusting for audiometric hearing levels. The limited prior research investigating associations between speech-in-noise performance and cognitive decline demonstrates inconsistent results. One study conducted among 837 participants (Mean baseline age = 65 years) from the Rotterdam Study found baseline differences in cognitive performance, but no significant differences in rates of change associated with speech-in-noise performance after a mean follow-up of 4.4 years (12); another study reported associations between worse baseline word recognition in competing messages and faster decline in Trail Making Test Part B over 10-year follow-up among 1,274 participants aged 49 years at baseline in the Beaver Dam Offspring Study (13). The inconsistencies might result from characteristics of the study participants and different cognitive tests and speech-in-noise tests (recognizing digit triplets/words instead of sentences in noise) used.

When comparing the results regarding audiometric hearing and speech-in-noise performance, baseline differences in cognitive performance were only found for speech-in-noise performance. The observed associations between speech-in-noise performance and cognitive decline were driven by those with relatively intact speech perception function at baseline. Differences in the findings might reflect underlying brain pathologies not yet captured by audiometric hearing. Moreover, in terms of cognitive domains being impacted by these two hearing measures, both audiometric hearing and speech-in-noise performance are associated with accelerated decline in language, executive function and attention, but memory domain is significantly impacted by worse speech-in-noise performance instead of audiometric hearing. Though there is no well-supported hypothesis, memory, especially those aspects that required learning new materials, might decline earlier with advancing age (37). Audiometric hearing might fail to capture the decline in memory function in our study population with a mean age of 75 years. And speech-in-noise performance, as a surrogate marker of brain health, involves remembering and understanding sentences to repeat them back during the test, might thus be more sensitive to memory decline.

Our examination of interaction by age, sex and race showed an interaction between speech-in-noise performance and race in the memory domain, where Black participants showed accelerated decline in memory with worse speech-in-noise performance while White participants showed no difference in rates of decline. The observed differential associations among Black and White participants might be explained by health disparities across the lifespan. For example, White participants might have higher cognitive reserve due to positive psychosocial and lifestyle factors and are thus more resilient to cognitive aging and have compensatory strategies (38). However, our examination of interaction is still exploratory, and we cannot draw conclusions regarding interaction. More research is needed to examine differential relationships between hearing and cognition by demographic factors.

Our study has a number of strengths: First, in this well-established cohort of community-dwelling older adults, the comprehensive battery of neurocognitive tests measured over a mean follow-up time of 3.5 years enabled longitudinal investigation of global and domain-specific cognitive performance. Second, in addition to pure-tone audiometry that primarily measures peripheral auditory function, we also included speech-in-noise performance assessed using the QuickSIN test, which is a reliable measure of central auditory function. The QuickSIN test is commonly used in clinical settings and more applicable to clinical practices as it is easy and quick to administer. Additionally, we are able to capture both peripheral and central aspects of hearing and compare our findings, which can provide more insights into the underlying pathways. Last, in exploratory analysis, we investigated interactions by a set of demographic characteristics to investigate potential differences by sub-groups who might experience accelerated cognitive decline. Although our study still has a relatively limited sample size and follow-up period, we have expanded upon previous works in the BLSA cohort and added to the currently limited body of literature by investigating speech-in-noise performance and cognition longitudinally. Also, the BLSA cohort consists of healthy adults and our results might not be generalizable to the general U.S. older adult population. In addition, because magnetic resonance imaging (MRI) measures are associated with both hearing and cognition, future studies with MRI measures might consider the roles of brain structure and function.

In conclusion, our study demonstrated longitudinal associations of both audiometric hearing and speech-in-noise performance with accelerated decline in cognition among a sample of community-dwelling older adults. Moreover, participants with worse speech-in-noise performance, but not audiometric hearing loss, had worse baseline cognitive performance. Audiometric hearing, primarily as a measure of peripheral auditory function, might be a risk factor for cognitive decline and might be targeted to delay cognitive decline. Comparatively, speech-in-noise performance, as it involves higher-level cognitive processing, might be a novel risk marker of underlying cognitive aging and may be uniquely sensitive to decline in memory function, contributing to early identification of individuals more vulnerable to cognitive decline and might be an easy-to-use tool applicable to clinical settings. Future longitudinal studies with audiometric hearing and speech-in-noise performance are needed.

## Data availability statement

The data analyzed in this study was obtained from the Baltimore Longitudinal Study of Aging (BLSA), the following licenses/restrictions apply: Researchers can only access BLSA data after approval of an Analysis Plan. Requests to access these datasets should be directed to BLSA, https://blsa.nia.nih.gov/how-apply.

## Ethics statement

The studies involving human participants were reviewed and approved by the Institutional Review Board of the Intramural Research Program of the National Institutes of Health. The patients/participants provided their written informed consent to participate in this study.

## Author contributions

Study concept or design: NA, YA, AG, JS, FL, LF, SR, JD, and DP. Analysis or interpretation of data: KJ, DP, and JD. Drafting the manuscript: KJ and DP. Critical revision of the manuscript: NA, YA, AG, JS, FL, LF, SR, and JD. All authors contributed to the article and approved the submitted version.
