# Supplementary material for: Associations of audiometric hearing and speech-in-noise performance with cognitive decline among older adults: The Baltimore Longitudinal Study of Aging (BLSA)
Source: Front Neurol. 2022 Dec 9;13:1029851. doi: 10.3389/fneur.2022.1029851 (PMC9784219; doi:10.3389/fneur.2022.1029851)
Supplement: Supplementary file 1 [file Image_1.pdf]

SUPPLEMENTARY MATERIAL

Supplementary Figure 1. Associations of Audiometric Hearing With Cognitive Performance by Age (>75 vs. ≤ 75 years) in the Baltimore Longitudinal Study of Aging 2012-2019 (N=702). Linear mixed effects models with random intercept, random slope, unstructured covariance structure and robust standard errors. Models adjusted for sex, race, education, smoking, body mass index, depression, hypertension, diabetes and elevated cholesterol.

Supplementary Figure 2. Associations of Audiometric Hearing With Cognitive Performance by Sex (Males vs. Females) in the Baltimore Longitudinal Study of Aging 2012-2019 (N=702). Linear mixed effects models with random intercept, random slope, unstructured covariance structure and robust standard errors. Models adjusted for age, race, education, smoking, body mass index, depression, hypertension, diabetes and elevated cholesterol.

Supplementary Figure 3. Associations of Audiometric Hearing With Cognitive Performance by Race (White vs. Black) in the Baltimore Longitudinal Study of Aging 2012-2019 (N=656). Linear mixed effects models with random intercept, random slope, unstructured covariance structure and robust standard errors. Models adjusted for age, sex, education, smoking, body mass index, depression, hypertension, diabetes and elevated cholesterol.

Supplementary Figure 4. Associations of Speech-in-Noise Performance With Cognitive Performance by Age (>75 vs. ≤ 75 years) in the Baltimore Longitudinal Study of Aging 2012-2019 (N=702). Linear mixed effects models with random intercept, random slope, unstructured covariance structure and robust standard errors. Models adjusted for sex, race,

education, smoking, body mass index, depression, hypertension, diabetes, elevated cholesterol and better-ear pure-tone average.

Supplementary Figure 5. Associations of Speech-in-Noise Performance With Cognitive Performance by Sex (Males vs. Females) in the Baltimore Longitudinal Study of Aging 2012-2019 (N=702). Linear mixed effects models with random intercept, random slope, unstructured covariance structure and robust standard errors. Models adjusted for age, race, education, smoking, body mass index, depression, hypertension, diabetes, elevated cholesterol and better-ear pure-tone average.

Supplementary Figure 6. Associations of Speech-in-Noise Performance With Cognitive Performance by Race (White vs. Black) in the Baltimore Longitudinal Study of Aging 2012-2019 (N=656). Linear mixed effects models with random intercept, random slope, unstructured covariance structure and robust standard errors. Models adjusted for age, sex, education, smoking, body mass index, depression, hypertension, diabetes, elevated cholesterol and better-ear pure-tone average.

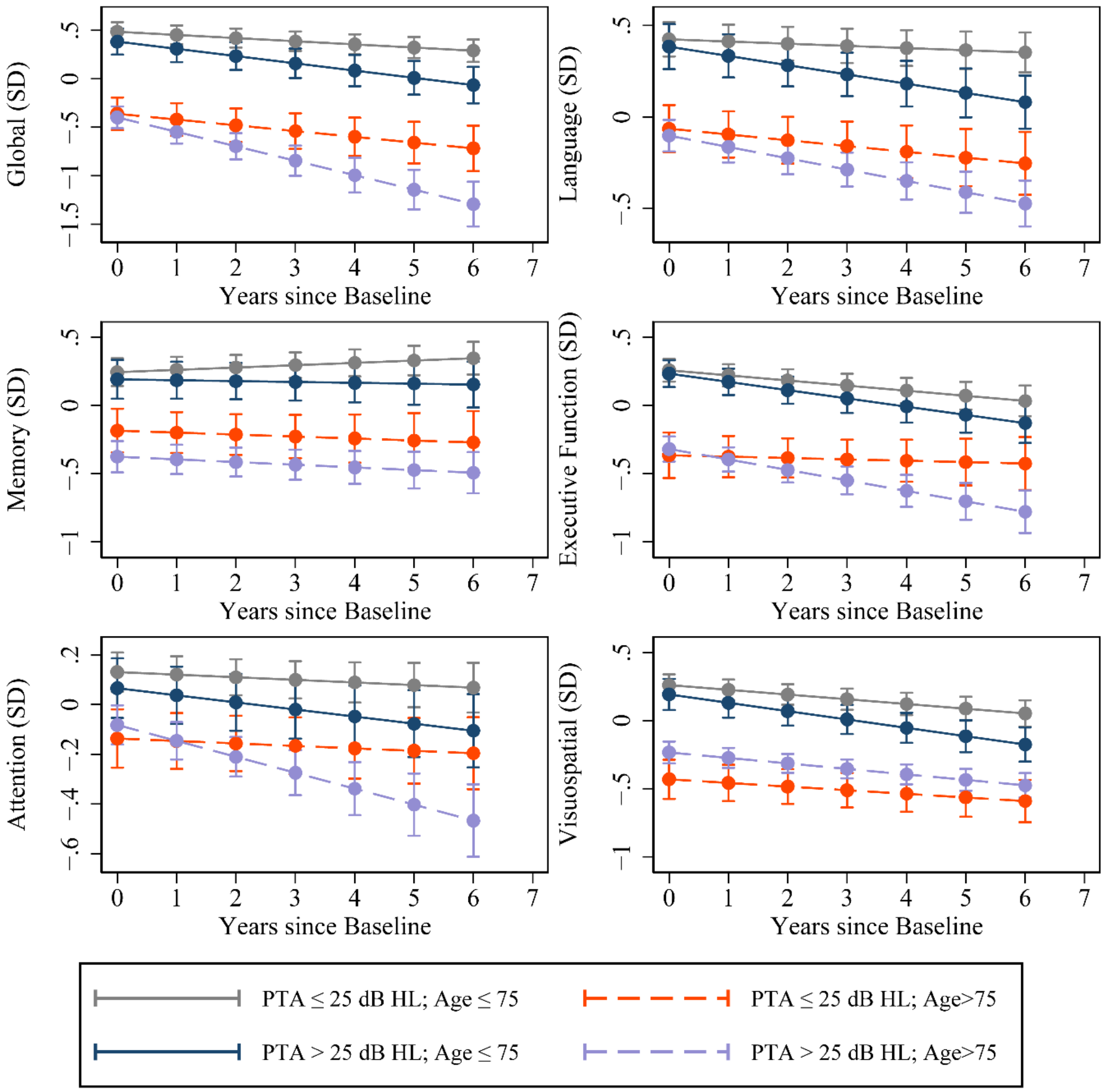

eFigure 1. Associations of Audiometric Hearing With Cognitive Performance by Age (>75 vs. ≤ 75 years) in the Baltimore Longitudinal Study of Aging 2012-2019 (N=702)

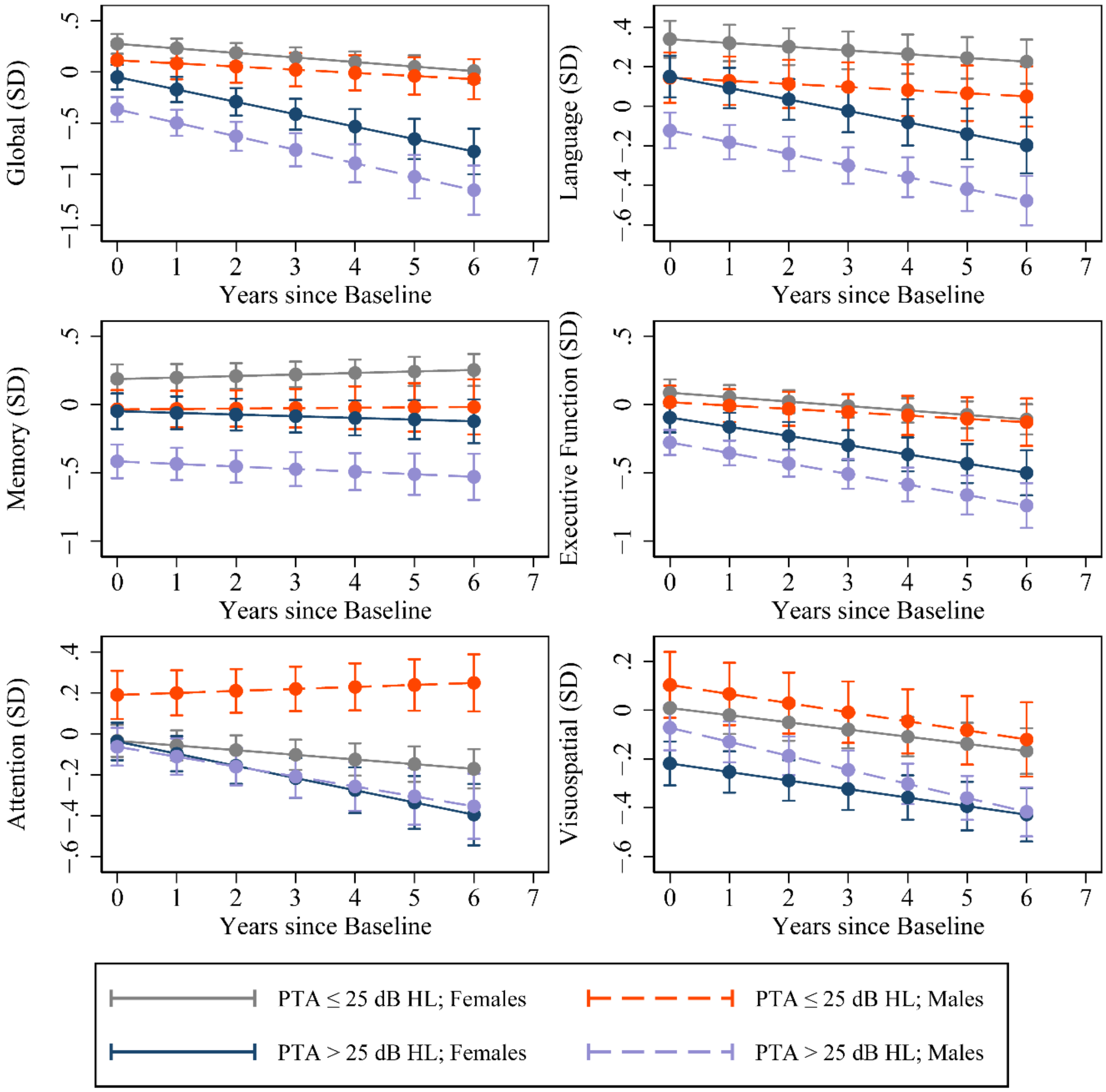

eFigure 2. Associations of Audiometric Hearing With Cognitive Performance by Sex (Males vs. Females) in the Baltimore Longitudinal Study of Aging 2012-2019 (N=702)

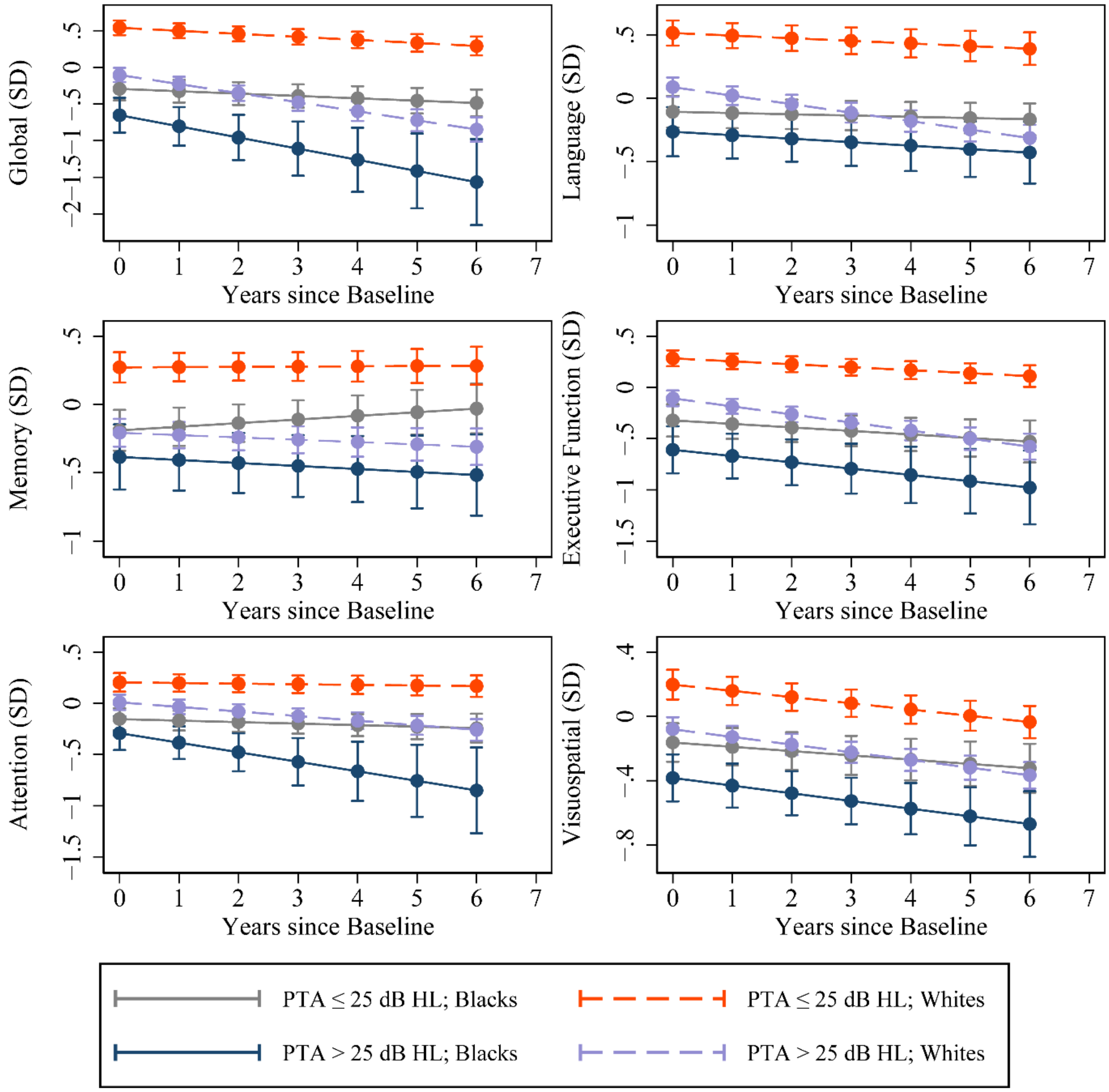

eFigure 3. Associations of Audiometric Hearing With Cognitive Performance by Race (White vs. Black) in the Baltimore Longitudinal Study of Aging 2012-2019 (N=656)

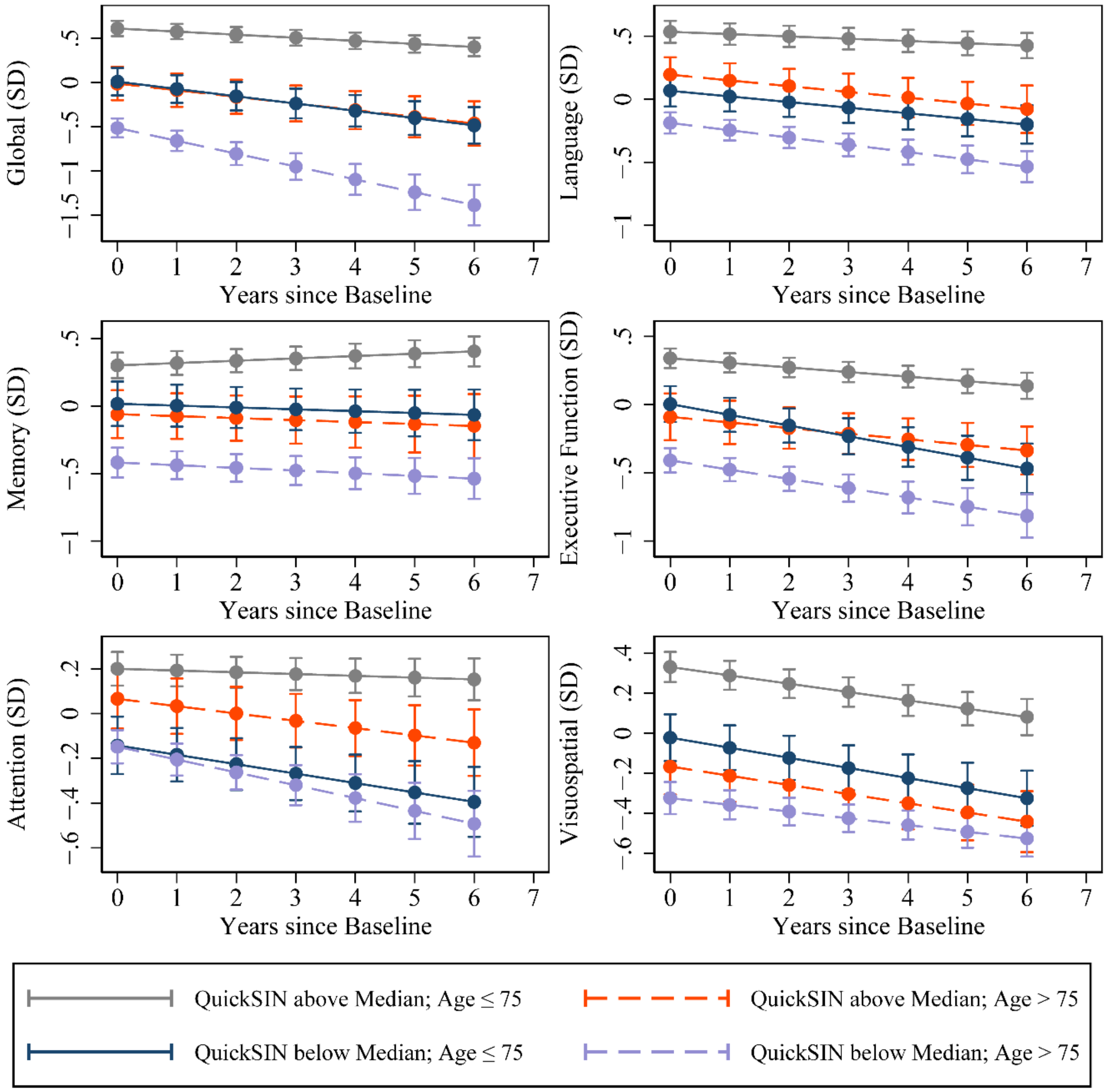

eFigure 4. Associations of Speech-in-Noise Performance With Cognitive Performance by Age (>75 vs. ≤ 75 years) in the Baltimore Longitudinal Study of Aging 2012-2019 (N=702)

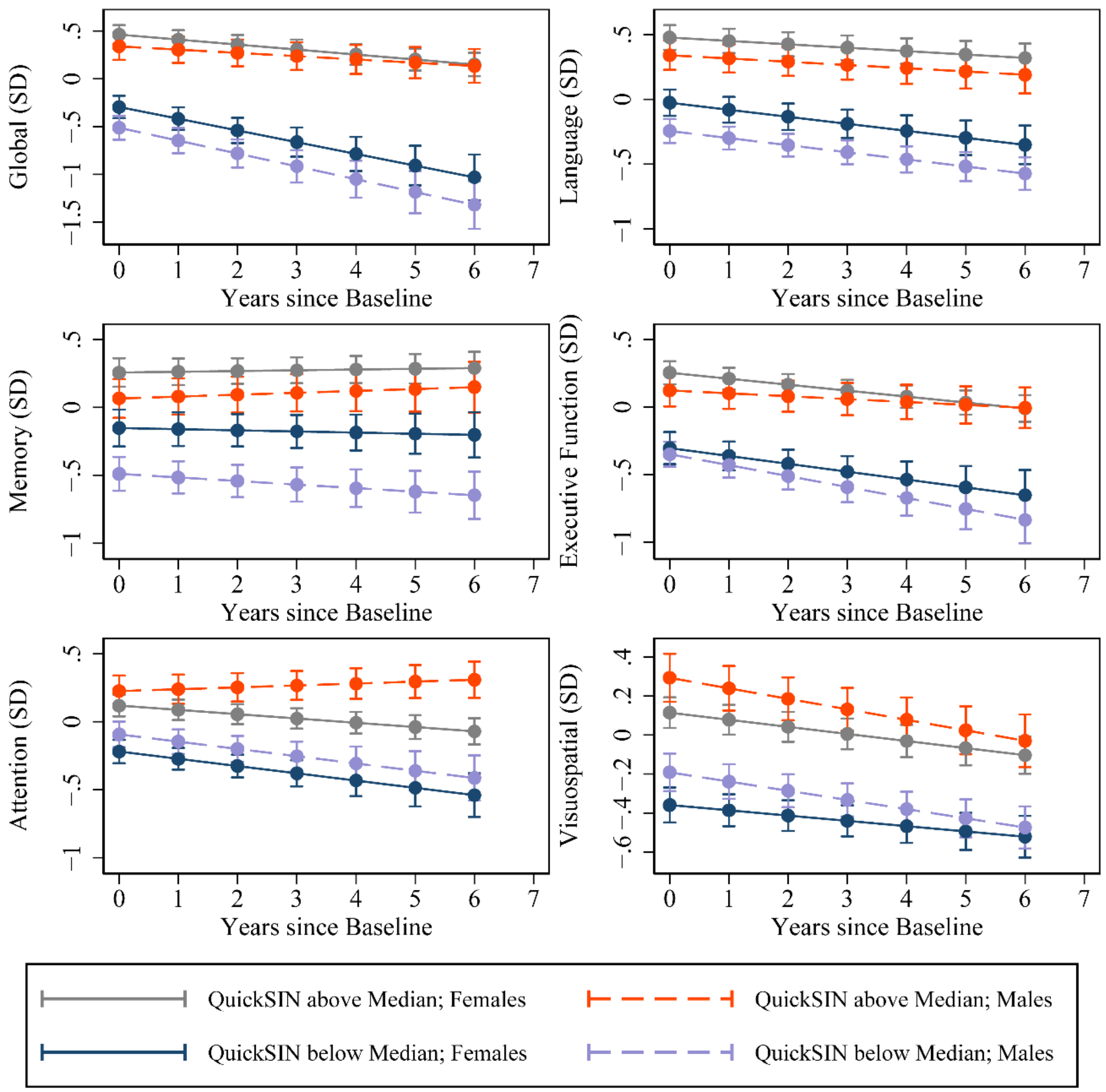

eFigure 5. Associations of Speech-in-Noise Performance With Cognitive Performance by Sex (Males vs. Females) in the Baltimore Longitudinal Study of Aging 2012-2019 (N=702)

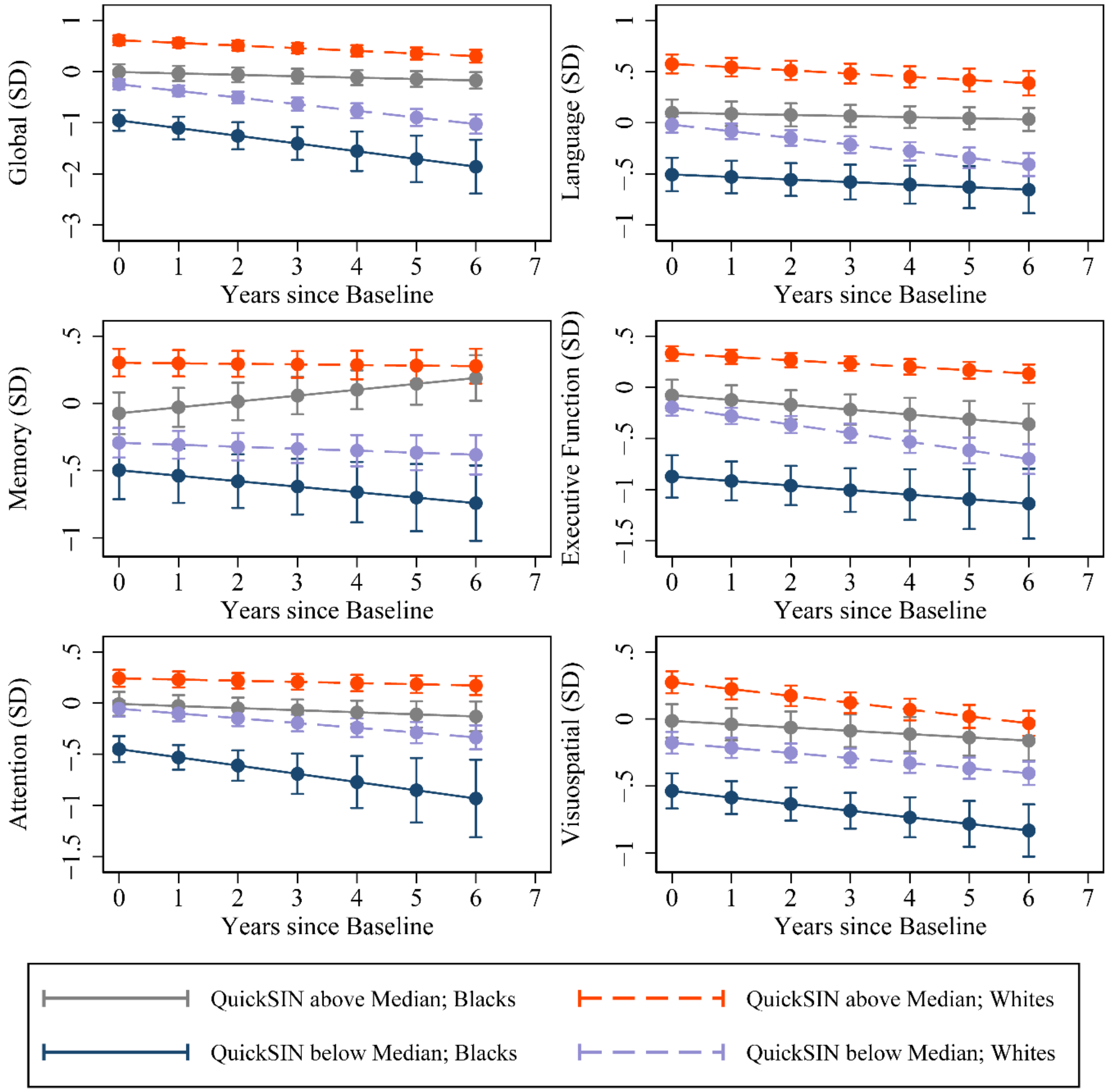

eFigure 6. Associations of Speech-in-Noise Performance With Cognitive Performance by Race (White vs. Black) in the Baltimore Longitudinal Study of Aging 2012-2019 (N=656)
